# Supplementary material for: Correlation between Microstructural and Magnetic Properties of Epitaxial YIG Films by Pulsed Laser Deposition
Source: ACS Omega. 2026 Feb 10;11(7):12612–21. doi: 10.1021/acsomega.5c12736 (PMC12947043; doi:10.1021/acsomega.5c12736)
Supplement: Supplementary file 1 [file ao5c12736_si_001.pdf]

Supporting Information: Correlation between microstructural and magnetic properties of epitaxial YIG films by pulsed laser deposition

José Diogo Costa[1,2,*], Niels Claessens[1], Giacomo Talmelli[1], Davide Tierno[1], Farah Amar[3,†], Thibaut Devolder[3], Matthijn Dekkers[4, ‡], Johan Swerts[1], Sean R.C. McMitchell[1], Florin Ciubotaru[1], and Christoph Adelmann[1,*]

[1]*Imec, 3001 Leuven, Belgium*

[2]*2Centro Singular de Investigación en Química Biolóxica e Materiais Moleculares (CIQUS), Universidade de Santiago de Compostela, 15782 Santiago de Compostela, Spain; Departamento de Química-Física, Universidade de Santiago de Compostela, 15782 Santiago de Compostela, Spain;*

[3]*Université Paris-Saclay, CNRS, Centre de Nanosciences et de Nanotechnologies, 91120 Palaiseau, France*

[4]*SolMateS BV, Auke Vleerstraat 3, 7521 PE Enschede, The Netherlands*

**Authors to whom correspondence should be addressed. Electronic mail: josediogo.teixeira@usc.es and christoph.adelmann@imec.be
†Present address: Keysight Technologies Inc. Santa Rosa, CA, US 95403
‡Present address: Lam Research Corporation, Fremont, CA, US 94538-0000

A. Structure and composition

To gain deeper insight into the nature of the defects observed at the highest deposition temperature (790 °C), a detailed scanning transmission electron microscopy (STEM) investigation was carried out. Figure S1 shows images of the same defect region captured using two complementary STEM imaging modes: annular bright-field (ABF) [Fig. S1(a)] and Z-contrast (ZC) [Fig. S1(b)]. The ABF mode, which is sensitive to both crystal structure and density variations, reveals pronounced contrast in the defect region, suggesting a structural difference relative to the surrounding crystalline lattice. In contrast, the ZC mode, which is primarily sensitive to atomic number and mass density, exhibits only weak contrast variations within the same area (highlighted by the red circle), indicating limited changes in local composition. These observations suggest that the defects mainly arise from local crystallinity changes, potentially accompanied by slight variations in stoichiometry.

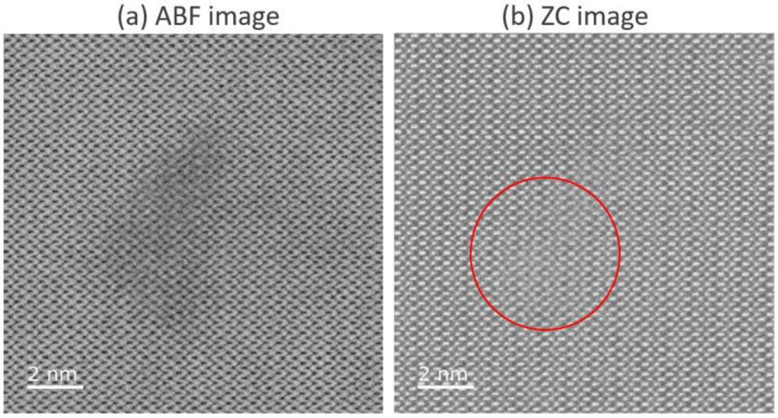

Fig. S1 – (a) ABF and (b) ZC STEM imaging of the defect region in the YIG film deposited at 790°C. The red circle indicates the position of the defect in the ZC image.

Additionally, atomic concentration profiles were measured by Energy-Dispersive X-ray Spectroscopy (EDS) for films deposited at both 650 °C and 790 °C to assess stoichiometric differences. As shown in Fig. S2, the profiles demonstrate a clear compositional variation between the two deposition temperatures, with a higher O content in the film deposited at 790 °C.

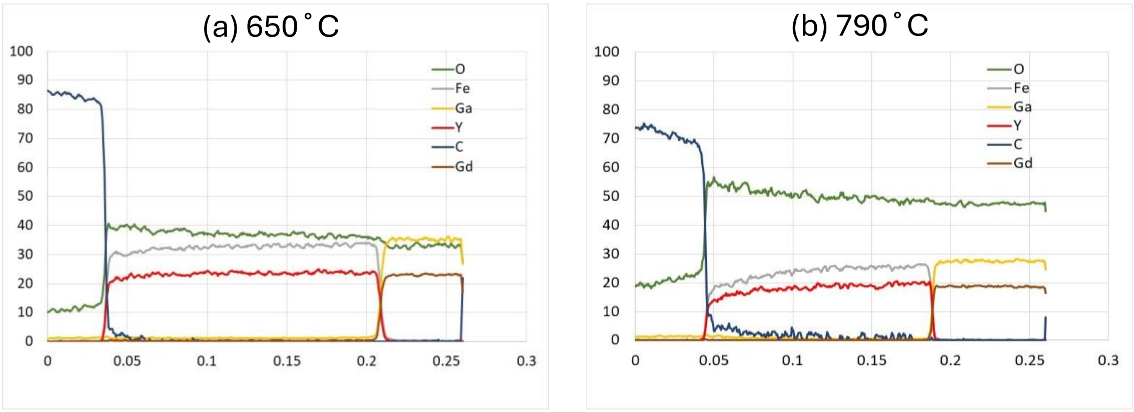

Fig. S2 – EDS atomic concentration profiles for YIG films deposited at (a) 650 °C, and (b) 790 °C.

This is supported by Rutherford Backscattering Spectrometry (RBS) and Elastic Recoil Detection (ERD) measurements, which yield stoichiometries shown below.

Deposition at 650 ˚C:

- Y: 3.7±0.07
- Fe: 4.9±0.1
- O: 12.4±0.2

Deposition at 790 ˚C:

- Y: 3.9±0.08
- Fe: 4.3±0.09
- O: 11.8±0.2

Thus, the observed defects correspond to regions with different crystallography and stoichiometry, which introduce local strain and degrade the overall crystalline quality of the film. The film stoichiometry was calculated by measuring the Y and Fe content using RBS and ERD analysis to determine the Y/O ratio. Figure S3 shows an example of the measured RBS and ERD measured spectra for the sample deposited at 650 ˚C. The error magnitudes were obtained from the 1-sigma absolute uncertainty extracted from the measurements.

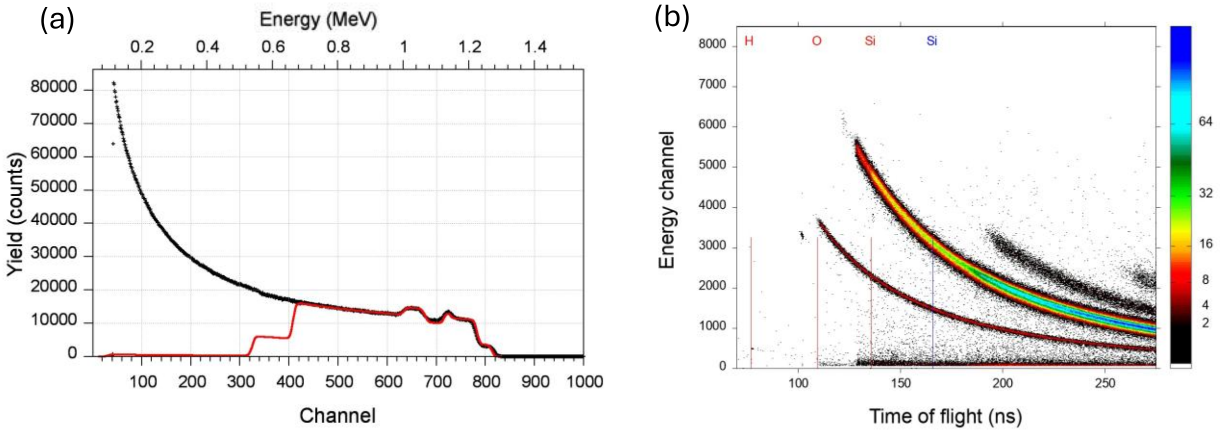

Fig. S3 – (a) RBS and (b) ERD measured spectra for the sample deposited at 650 ˚C.

B. Ferromagnetic Resonance

Vector network analyzer (VNA) ferromagnetic resonance (FMR) is an electrical characterization technique that allows for the characterization of a broad range of magnetization dynamics. It facilitates the extraction of key magnetic parameters, including the effective magnetization (M_{eff}) and the magnetic damping (α) [1].

Considering the Landau–Lifshitz–Gilbert (LLG) equation, which describes the damped precessional motion of magnetization, the following relations can be derived [2]:

$w_{FMR} \approx \gamma_0(H - M_{eff})$ and

$\Delta w \approx 2\alpha \cdot w_{FMR} + \gamma_0 H_0$,

where w_{FMR} denotes the FMR frequency, Δw the FMR linewidth, H is the external applied magnetic field, γ_0 is the gyromagnetic ratio and H_0 an equivalent field representing the inhomogeneous (frequency independent) linewidth broadening. $M_{eff} = M_s - H_k$ is the effective magnetization, which is comprised by the saturation magnetization (M_s) and an out-of-plane

anisotropy field (H_k) that may originate from magnetocrystalline or magnetoelastic contributions. It is clear that α and M_{eff} can be determined by fitting Δw as a function of w_{FMR}, and w_{FMR} as a function of H, respectively.

Figure S4 shows the fitting results of the VNA-FMR measurement data for the sample deposited with a fluence of 1.1 J/cm^2, deposition pressure of 0.1 mbar, deposition temperature of 650 °C, and annealing temperature of 900 °C, representative of other low-damping YIG films. In Fig. S4(a), a typical Lorentzian fit is displayed, showing excellent agreement with the measured data and yielding a negligible fitting error. This behaviour was observed even in a non-ideal (bi-phase) case, which was deliberately selected to demonstrate the robustness of the fitting procedure. Systematic errors were further minimized through linear regression, and poorly fitted data points (outliers) were excluded following a thorough statistical analysis.

The linear relationship between frequency and field enables the direct extraction of M_{eff} from the frequency-field dependence. Figure S4(b) exhibits a significant slope (in the GHz range), resulting in a low relative error. Even for low-signal samples, the statistical uncertainty remains below 1%. In contrast, for the determination of the Gilbert damping constant α, the linewidth-frequency relationship [Fig. S4(c)] shows a much smaller slope (in the MHz range), leading to a noticeably higher uncertainty. Nonetheless, the statistical error in α remains below 5% ($\Delta\alpha \sim 10^{-5}$), which is sufficient for accurate characterization of state-of-the-art low-damping YIG films.

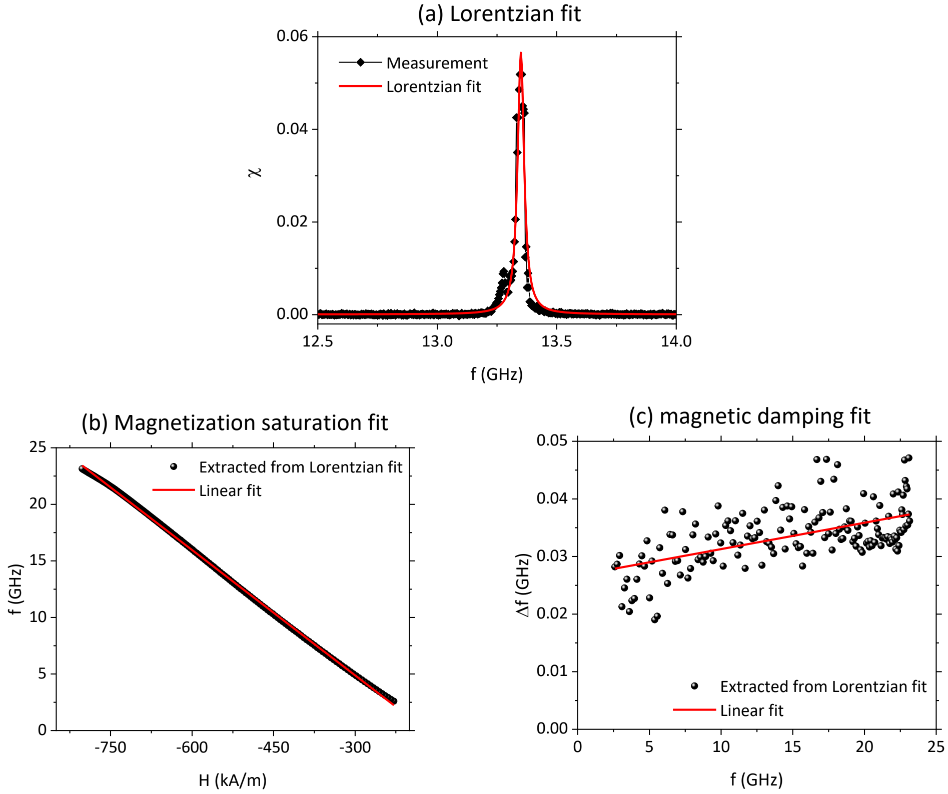

Fig. S4 – FMR analysis. (a) VNA measurement (black dots) and Lorentzian fit (red line) obtained for sample 2 at a magnetic field of 1010 kA/m. (b) w_{FMR} as a function of the magnetic field to extract M_{eff}. (c) Δw as a function of the frequency to extract α.

However, it is important to account for potential sources of error introduced by the VNA measurement itself. A critical initial step in FMR characterization is the VNA calibration. Imperfect calibration can introduce a residual phase shift in the measurement, leading to a mixing of the real (*Re*) and imaginary (*Im*) components of the reflection coefficient S_{11}. In practice, some calibration error is inevitable, resulting in some intermixing. This artifact can distort the measured signal and is mathematically equivalent to a linear transformation of the real and imaginary components, as described by:

$$Im(S_{11}) = (1-x)Im(S_{11}) + xRe(S_{11}),$$

$$Re(S_{11}) = (1-x)Re(S_{11}) + xIm(S_{11}),$$

Here, x is the fraction of intermixing. In the following, we consider a virtual signal given by [2]:

$$S_{11} = e^{-i2l\frac{\omega}{c}\sqrt{\widetilde{\varepsilon_r}}} e^{-i2l\frac{\omega}{c}\sqrt{\widetilde{\varepsilon_r}}\frac{1}{2}x\chi},$$

with

$$\chi = \frac{M_s H_{eff,z}}{H^2 - \left(\frac{\omega}{\gamma_0}\right)^2 + 2i\alpha H \frac{\omega}{\gamma_0}}.$$

For consistency, the parameters α, M_s, and γ_0 were taken from the sample analyzed in Fig. S4. The relative permittivity $\widetilde{\varepsilon_r}$ corresponds to that of the GGG substrate, and l = 50 μm denotes the length of the coplanar waveguide used in the measurement setup. A value of x = 1% was assumed as a reasonable estimate to model the phase shift-induced mixing of the real and imaginary components of the S_{11} parameter. Based on this idealized scenario—considering only the intermixing parameter—we estimate a resulting measurement error of approximately 2×10^{-4}, which represents the idealized FMR signal free from statistical error. Based on this approach, we estimate the resulting measurement error to be approximately 2×10^{-4}.

It is important to emphasize that this estimation is based on simulated or "virtual" data, making it independent of any specific experimental setup. To minimize the error, high-precision VNA calibration is essential. In our simulation, we assume a 1% intermixing between the real and imaginary parts, a level that, based on our experience, is realistic. Additionally, the CPW length, which directly influences the S_{11} response, contributes to the overall measurement error. Notably, most reported damping values for state-of-the-art YIG films fall in the range of 1-2 × 10^{-4} [3], [4], which is close to the error threshold we estimate. Reported values significantly below this, such as the record low value of 6×10^{-5}, become more contentious, particularly when derived using limited data points and without mentioning the CPW length, which could result in a statistical error comparable to the measured damping itself [5].

C. Vibrating sample magnetometry

Vibrating sample magnetometry (VSM) was also performed to complement the magnetic analysis using FMR. The VSM measurements confirm the presence of an out-of-plane (OOP) magnetization component (Fig. S5). The data further show that this OOP component increases with rising M_{eff}, as illustrated for $M_{eff} = 168kA/m$ [Fig. S5(a)] and $M_{eff} = 177kA/m$ [Fig. S5(b)], values extracted by FMR measurements. Suggesting that the enhancement of M_{eff} is associated with a more pronounced OOP component as film distortion increases [see Fig. 3(e) of the article]. However, due to the low magnetic signal of the ferrimagnetic material and the diamagnetic contribution of the sample holder, the absolute magnetization could not be reliably extracted from the VSM measurements.

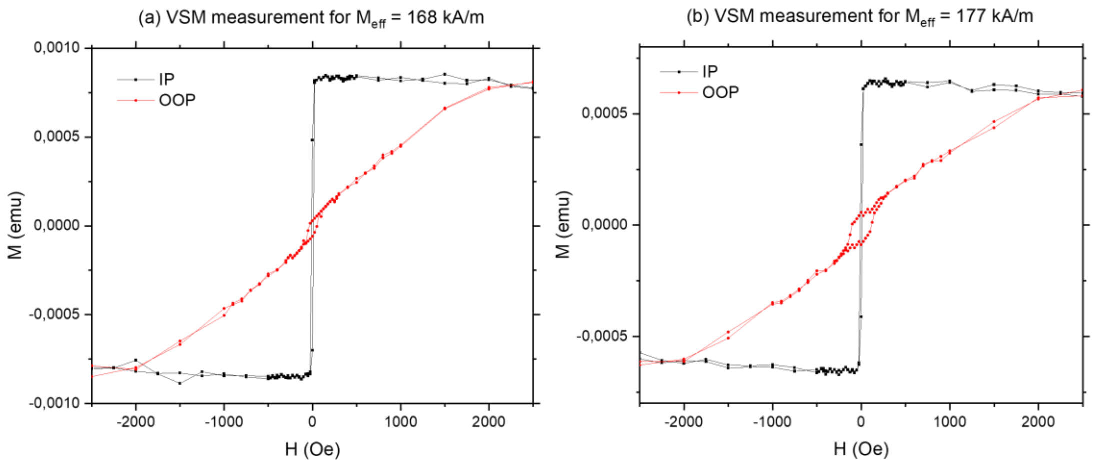

Fig. S5 – VSM magnetic measurement in the in-plane (black line) and out-of-plane directions (red line) for the samples with (a) M_{eff} = 168 kA/m and (b) M_{eff} = 177 kA/m (M_{eff} from FMR measurements).

Supporting Information

Annular bright-field (ABF) and Z contrast (ZC) STEM imaging of the defect region in the YIG film deposited at 790°C (Figure S1). Energy-Dispersive X-ray Spectroscopy (EDS) atomic concentration profiles for YIG films deposited at 650 °C, and 790 °C (Figure S2). Rutherford Backscattering Spectrometry (RBS) and Elastic Recoil Detection (ERD) measured spectra for the sample deposited at 650˚C (Figure S3). Vector Network Analyzer Ferromagnetic Resonance (VNA-FMR) analysis (Figure S4). Vibrating sample magnetometry (VSM) measurement in the in-plane and out-of-plane directions (Figure S5).

References

[1] S. Tamaru, S. Tsunegi, H. Kubota, and S. Yuasa, “Vector network analyzer ferromagnetic resonance spectrometer with field differential detection,” *Rev. Sci. Instrum.*, vol. 89, no. 5, 2018.

[2] T. Devolder, “Using rf voltage induced ferromagnetic resonance to study the spin-wave density of states and the Gilbert damping in perpendicularly magnetized disks,” *Phys. Rev. B*, vol. 104413, pp. 1–11, 2017.

[3] M. C. Onbasli; A. Kehlberger; D. H. Kim; G. Jakob; M. Kläui; A. V. Chumak; B. Hillebrands; C. A. Ross, “Pulsed laser deposition of epitaxial yttrium iron garnet films with low Gilbert damping and bulk-like magnetization,” *APL Mater.*, vol. 2, no. 106102, 2014.

[4] M. B. Jungfleisch, A. V. Chumak, A. Kehlberger, V. Lauer, D. H. Kim, M. C. Onbasli, C. A. Ross, M. Kläui, and B. Hillebrands, “Thickness and power dependence of the spin-pumping effect in Y3Fe5O12/Pt heterostructures measured by the inverse spin Hall effect,” *Phys. Rev. B*, vol. 91, no. 134407, p. 134407, Apr. 2015.

[5] Christoph Hauser, Tim Richter, Nico Homonnay, Christian Eisenschmidt, Mohammad Qaid, Hakan Deniz, Dietrich Hesse, Maciej Sawicki, Stefan G. Ebbinghaus and Georg Schmidt, “Yttrium Iron Garnet Thin Films with Very Low Damping Obtained by Recrystallization of Amorphous Material,” *Sci. Rep.*, vol. 6, p. 20827, 2016.
